# Actinic Keratosis Clinical Practice Guidelines: An Appraisal of Quality

**DOI:** 10.1155/2015/456071

**Published:** 2015-09-16

**Authors:** Joslyn S. Kirby, Thomas Scharnitz, Elizabeth V. Seiverling, Hadjh Ahrns, Sara Ferguson

**Affiliations:** ^1^Department of Dermatology, Penn State Milton S. Hershey Medical Center, Hershey, PA 17033, USA; ^2^Penn State College of Medicine, Hershey, PA 17033, USA; ^3^Department of Family and Community Medicine, Penn State Milton S. Hershey Medical Center, Hershey, PA 17033, USA

## Abstract

Actinic keratosis (AK) is a common precancerous skin lesion and many AK management guidelines exist, but there has been limited investigation into the quality of these documents. The objective of this study was to assess the strengths and weaknesses of guidelines that address AK management. A systematic search for guidelines with recommendations for AK was performed. The Appraisal of Guidelines for Research and Evaluation (AGREE II) was used to appraise the quality of guidelines. Multiple raters independently reviewed each of the guidelines and applied the AGREE II tool and scores were calculated. Overall, 2,307 citations were identified and 7 fulfilled the study criteria. The Cancer Council of Australia/Australian Cancer Network guideline had the highest mean scores and was the only guideline to include a systematic review, include an evidence rating for recommendations, and report conflicts of interest and funding sources. High-quality, effective guidelines are evidence-based with recommendations that are concise and organized, so practical application is facilitated. Features such as concise tables, pictorial diagrams, and explicit links to evidence are helpful. However, the rigor and validity of some guidelines were weak. So, it is important for providers to be aware of the features that contribute to a high-quality, practical document.

## 1. Introduction

Actinic keratosis (AK), or solar keratosis, is a common dysplastic lesion of the keratinocyte [1, 2]. Over a 10-year period from 1990 to 1999, AK was diagnosed in more than 47 million visits and was found to occur in 14% of all patients visiting dermatologists [3]. AK may be treated due to concern for carcinogenesis, patient discomfort, or cosmesis. The most common treatment for AK is cryotherapy [4], likely due to easy access, ease of care for patients, and speed of the procedure. Other management methods include observation, alternative destructive therapies, topical chemotherapies, chemical peels, photodynamic therapy (PDT), and preventative measures such as sunscreen [5]. Given the high prevalence of AK and the variety and number of clinicians within an organization that encounter AK, CPG can serve as a resource upon which management approaches are unified.

Clinical practice guidelines (CPG) can guide clinicians' diagnostic and therapeutic decisions by succinctly reviewing the literature and proposing evidence-based management recommendations. CPG are defined by the Institute of Medicine as “statements that include recommendations intended to optimize patient care that are informed by a systematic review of evidence and an assessment of the benefits and harms of alternative care options. Rather than dictating a one-size-fits-all approach to patient care, CPG offer an evaluation of the breadth and quality of the relevant scientific literature and an assessment of the likely benefits and harms of a particular treatment” [6]. Thus, a high-quality CPG will include an extensive literature review and an evidence rating scale can help readers be aware of the potential limitations of the sources. The quality of CPG can vary for many reasons including the adequacy of the literature search, types of studies incorporated, and bias by the authors [7, 8]. To our knowledge, there is no published assessment of AK CPG quality. We used the AGREE II instrument [9] to assess the strengths and weaknesses of CPG addressing multiple modalities of AK management for patients without immunosuppression.

## 2. Methods

The PRISMA checklist was used as a guide for this investigation. A medical librarian performed a systematic search of the medical literature for CPG with recommendations for AK. An extensive search of Medline/PubMed (1966–March 20, 2014) was conducted for relevant clinical practice guidelines using a combination of the key terms “actinic” and “keratosis” and “guideline” as well as “keratosis, actinic” as a major medical subject heading. Results were limited to practice guideline and guideline publication types. There were no date or language restrictions. In addition the National Guideline Clearinghouse, National Institute for Health and Clinical Excellence, European Academy of Dermatology and Venereology, Guidelines International Network, TRIP Database, Australian Government Clinical Practice Guidelines Portal, Scottish Intercollegiate Guidelines Network, Guidelines and Audit Implementation Network, Professional Organizations and Royal Colleges, Health Service Executive Guidelines and the American Academy Dermatology web sites were searched using key terms “actinic” and “keratosis.”

The inclusion criteria were established* a priori* and (1) included an explicit statement identifying the document as a management guideline, (2) were written by multiple authors including at least one dermatologist, and (3) made recommendations concerning multiple options to manage AK. Exclusion criteria included (1) consensus statements or review articles that did not include identifiable management recommendations, (2) reviews of a published guideline, (3) focus on only preventative, epidemiology, or research methods, (4) focus on a limited or specific patient population, and (5) discussion of one form of management.

Two reviewers (TS and JK) independently examined the retrieved titles and abstracts to assess the articles for inclusion and exclusion criteria. The full text of these articles was retrieved and the same two reviewers (TS and JK) reviewed the selected articles again for eligibility. Disagreement was resolved through discussion by the reviewers (TS and JK). Seven of the guidelines were selected for analysis.

The AGREE II tool was used to describe the quality of the selected CPG. It was developed to evaluate the validity and feasibility of CPG as well as assess sources of bias [9]. The AGREE and AGREE II tools have been validated and widely applied, including CPG of dermatologic conditions [17, 18] as well as in other disciplines [19–21]. Four of the authors independently reviewed and scored the CPG. JK and ES are dermatologists, TS was a first-year medical student, and HA is a family practitioner. All four reviewers read the AGREE II users' manual and completed the online orientation and training.

The AGREE II instrument was used to define the assessment variables and rating system and to store data [9]. This instrument provides criteria to appraise the quality of CPG and consists of 23 items grouped into six domains: (1) scope and purpose, (2) stakeholder involvement, (3) rigor of development, (4) clarity of presentation, (5) applicability, and (6) editorial independence. Each item is rated on a seven-point Likert scale from strongly disagree to strongly agree (1–7, resp.). The results for each domain consist of a domain score, which is the sum of the scores of the individual domain items and standardized by scaling the total as a percentage of the maximum possible score for that domain. The maximum score for each domain was the number of questions multiplied by 7 (strongly agree). The minimum score was the number of questions multiplied by number 1 (strongly disagree):

(1) Scaled Domain Score=Sum of Domain scores−Minimum possible scoreMaximum possible score−Minimum possible score×100%.

The minimum standardized score for each domain was, therefore, 0% and the maximum was 100%. A standardized domain score above 60% has been suggested as the threshold to indicate sufficient minimum quality to consider practical use of this portion of the guideline [18]. This study was exempted from review by the Penn State Institutional Review Board.

### 2.1. Statistical Analysis

The scores were compiled and reviewed; upon visual inspection one investigator had scores that appeared to deviate from the other three. The Pearson correlation coefficient is very sensitive to outlying values so Spearman's Rho was used to determine interrater reliability [22]. The degree of agreement was classified as follows: poor (0.00), slight (0.00 and 0.20), fair (0.21 to 0.40), moderate (0.41 to 0.60), substantial (from 0.61 to 0.80), and very good or almost perfect (0.81 to 1.00) [23]. A descriptive statistical analysis of the scores was performed and included the mean and standard deviation. A *p* value of <.05 was considered significant. All analyses were performed using SAS 9.3 (SAS, Cary, NC).

## 3. Results

**Figure 1:**
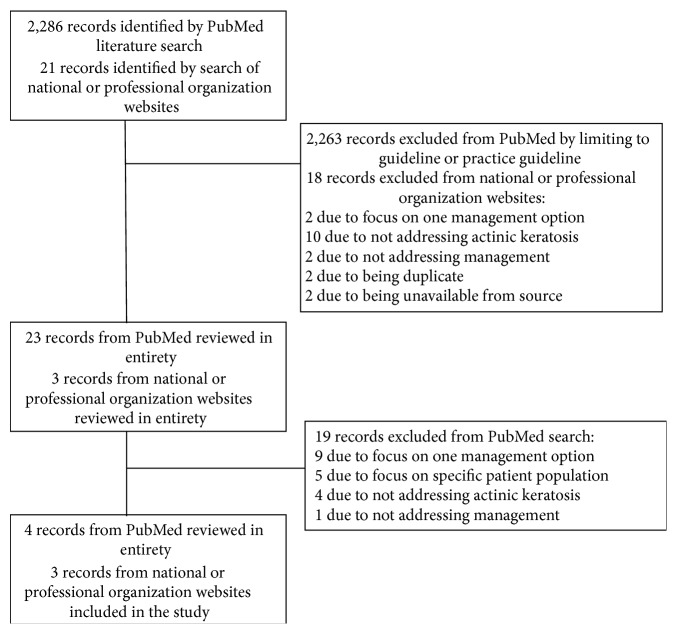
Literature search and study selection process.

The search strategy identified 2,307 records and after review ultimately seven records were included in the study (Figure 1). The characteristics of the seven CPG are listed in Table 1. Of the seven CPG, one was from Australia [11], one was from Britain [12], three were from Germany [13–15], one was from Italy [16], and one was from the United States [10]. The range for interrater agreement was 0.39–0.86. Analysis showed that one appraiser had only fair agreement with other appraisers (0.39 [*p* = .38]). This rater was a first-year medical student and it was presumed that this reflected clinical experience rather than CPG quality so his scores were removed and subsequent calculations did not include these scores. The correlation coefficients among the three remaining appraisers, which included two dermatologists and a family practitioner, were moderate or better (0.54–0.86).

The CCA/ACN guideline [11] was the only CPG to perform a systematic review, include evidence-based ratings for recommendations, and report conflicts of interest and funding sources (Table 1). It was also the only CPG to have scores for all domains above 60% and had the highest score for each of the six domains and overall category (Table 2). The domains with the highest mean scores were domain 1 (scope and purpose) and domain 4 (clarity of presentation) with means of 50.2% and 51.1%, respectively. The domains with the lowest mean scores were domain 5 (applicability) and domain 6 (editorial independence) with means of 28.6% and 32.1%, respectively. Domain 6 (editorial independence) also had the lowest individual score of 6.3 and the greatest range in individual scores (8.3% to 80.6%). This may reflect wide variation in disclosure of funding and conflict of interests. While five of the seven CPG included a conflict of interests statement, only two had a disclosure regarding funding sources (Table 1).

Three guidelines were recommended by at least three raters (CCA/CAN [11], de Berker et al. [12], Stockfleth and Kerl 2006 [13]). The CPG by Stockfleth et al. 2008 [14] was recommended by two of the three raters. This second article by Stockfleth et al. 2008 [14] had higher scores in domains 1 (scope and purpose), 2 (stakeholder involvement), 4 (clarity of presentation), and 5 (applicability) but lower scores in domains 3 (rigor of development) and 6 (editorial independence). Domain 3's (rigor of development) score may have been lower due to a lack of a systematic review and the methods which were reported as “consultation and discussion of best practice was adopted as a means of formulating a consensus of opinion.” This article reported funding by a pharmaceutical company that has AK-related products in its portfolio, which may have negatively impacted the scores in domain 6 (editorial independence). The 2011 update by this group (European Dermatology Forum) was recommended by two of the three raters and compared to the CPG from 2006 and 2008; this document had lower scores than the 2006 and 2008 CPG from this group across all domains except for domain 6 (editorial independence). In contrast to the two prior versions, this CPG had a table at the beginning of the document that listed the COI for each author; however there was no explicit statement of funding.

## 4. Discussion

High-quality CPG can supply clinicians with succinct research findings, specific suggestions for management in particular clinical scenarios, and economic considerations for a specific condition [24]. AK is a common skin lesion and many CPG have been published. Our goal was to review the quality of CPG that are broadly applicable to AK management rather than focus on a specific population or treatment. AKs are treated by providers from multiple disciplines with an array of treatments, so it is valuable and increasingly common for CPG to be written by representatives from the stakeholder groups, including generalists, specialists, and patients [8, 25]. The CCA/ACN was the only guideline to score above 60% for the stakeholder involvement domain; it was developed by a multidisciplinary “working party” that included dermatologists, pathologists, plastic surgeons, general practitioners, health economists, epidemiologists, and patients.

The CCA/CAN was also the only CPG that utilized a systematic review and had the highest scores for the rigor of development domain. The results of this review are consistent with previous appraisals of CPG for acne, psoriasis, and pressure ulcers that showed that the rigor of development domain often had lower scores [17, 18]. This domain is important to CPG value since it supports the validity of the recommendations [8]. Other CPG had less rigorous search methods or incomplete descriptions of the methods. CPG help readers to understand the strengths and limitations of the recommendations by including a rating of the supporting evidence. The CCA/ACN, Stockfleth and Kerl 2006 [13], and Stockfleth et al. 2011 [15] CPG used a published evidence-based rating scale, while the de Berker et al. [12] CPG used a rating scale based on evaluator opinion.

CPG rigor is also supported by an evaluation of potential sources of bias, such as conflict of interests (COI). The editorial independence domain includes a rating of COI and funding sources and had the lowest mean score of all the domains. COI reporting by the CPG contributors varied greatly. The Stockfleth et al. 2008 [14] article does address COI and discloses a grant that was received but failed to divulge that the grant came from a pharmaceutical group that produces topical fluorouracil and topical diclofenac products. The CCA/ACN and Stockfleth et al. 2011 [15] CPG clearly listed COI. Other articles (Rossi et al., Drake et al.) [10, 16] lacked a COI statement. The clearly and specifically worded COI and funding statements offered in the CCA/ACN guideline likely contributed to its having the highest mean score in this domain.

One of the primary purposes of CPG is to make practical, evidence-based recommendations about management of a condition. A summary of the recommendations from the three CPG with the highest scores is presented in Table 3. All seven CPG addressed common topical and destructive therapies. In addition, sun avoidance and sun-protection were included in all CPG as a part of AK prevention and management. Three CPG [11, 12, 16] summarized evidence that AKs have a low rate of malignant transformation, but only one [12] mentioned observation, emollients, and sunscreen as possible management options [26].

The results of this study should be considered in the context of the limitations, including CPG that were not included and the effects of score variation between raters and within raters. Since the literature search was completed in 2014, this review does not include CPG published more recently which may be valuable [27, 28]. This review included four reviewers but the scores for one reviewer were not used because they did not correlate well with the others. Also, the CPG were appraised according to the information contained within them and additional supporting documents were not evaluated. It is possible that additional information may have increased scores for some of the CPG but was simply missing from the published version of the guidelines. Also, it is important to consider that high-quality methods or well-written CPG do not necessarily produce valid recommendations.

Effective CPG are evidence-based with recommendations that are concise and organized, so practical application is facilitated [29–31]. The CCA/ACN guideline had the highest scores and included a clear, organized, and concise table at the beginning of the document with helpful evidence-linked recommendations. However, the reviewers found that the recommendations lacked sufficient detail to apply in a clinical setting. For example, the recommendations included “cryotherapy achieves consistently high cure rates for solar keratosis” but lacks details that would facilitate use, such as the duration of the freeze-thaw cycle or number of freeze-thaw cycles. Also, the CCA/ACN AK recommendations were interspersed among the recommendations for nonmelanoma skin cancer. This made identifying the AK-specific information challenging. Stockfleth et al. [14] included a pictorial algorithm and raters thought this was an effective method to present information, yet scores in other domains lagged behind the CCA/ACN CPG. In closing, CPG are useful tools to guide clinical practice based on existing evidence; however it is important for providers and guideline authors to be aware of the features that contribute to a high-quality, practical, and valuable document.

## Figures and Tables

**Table 1:** Characteristics of the included studies.

Guideline	Systematic review performed	Evidence grading reported	CPG funding reported	Competing interests reported
Drake et al. [10]	NR	No	NR	NR
CCA/ACN [11]	Yes	Yes	Yes	Yes
de Berker et al. [12]	No	Yes	NR	Yes
Stockfleth and Kerl (2006) [13]	NR	Yes	NR	Yes
Stockfleth et al. (2008) [14]	NR	No	Yes	Yes
Stockfleth et al. (2011) [15]	NR	Yes	NR	Yes
Rossi et al. [16]	NR	No	NR	NR

CCA/ACN: Cancer Council Australia/Australian Cancer Network. NR = not reported.

**Table 2:** Clinical practice guideline scores.

Guideline	Mean scores (% [SD])						Overall assessment (%, [SD])	Raters that would recommend, *n* = 3 raters	
	(1) Scope and purpose	(2) Stakeholder involvement	(3) Rigor of development	(4) Clarity of presentation	(5) Applicability	(6) Editorial independence		Yes	Yes, with modifications
Drake et al. [10]	33.3 [11.1]	35.2 [8.5]	16.7 [7.3]	31.5 [8.5]	6.9 [4.8]	11.1 [12.7]	5.6 [9.6]	0	0
CCA/CAN [11]	81.5 [3.2]	96.3 [3.2]	92.6 [20.1]	75.9 [23.1]	68.1 [18.8]	80.6 [12.7]	77.8 [9.6]	2	1
de Berker et al. [12]	35.2 [21.0]	31.5 [30.6]	52.8 [11.1]	70.4 [13.9]	31.9 [23.7]	33.3 [36.3]	38.9 [25.5]	1	2
Stockfleth and Kerl (2006) [13]	53.7 [17.9]	29.6 [3.2]	69.4 [18.2]	29.6 [13.9]	20.8 [11.0]	38.9 [19.2]	27.8 [25.5]	1	2
Stockfleth et al. (2008) [14]	87.0 [8.5]	51.9 [32.6]	47.2 [12.1]	59.3 [21.0]	31.9 [8.7]	8.3 [8.3]	27.8 [9.6]	0	2
Stockfleth et al. (2011) [15]	24.1 [8.5]	16.7 [0]	35.2 [8.0]	35.2 [11.6]	13.9 [2.4]	44.4 [4.8]	33.3 [16.7]	0	2
Rossi et al. [16]	37.0 [28.0]	18.5 [17.9]	24.1 [11.2]	55.6 [30.9]	26.4 [8.7]	8.3 [14.4]	22.2 [25.5]	0	1

Mean score for domain	50.2 [27.2]	39.9 [30.3]	48.3 [27.4]	51.1 [24.1]	28.6 [21.7]	32.1 [29.1]	33.3 [26.4]		

SD = standard deviation.

**Table 3:** Samples of recommendations from the three CPG with the highest overall scores.

Guideline	Samples of CPG recommendations
CCA/ACN [11]	(i) In many cases solar keratosis [AK] regresses spontaneously and uncommonly; it evolves into squamous cell carcinoma (ii) The chances that an individual solar keratosis [AK] will develop into SCC are extremely small; however when one encounters SCC, the chance that it has arisen in association with solar keratosis is very high (iii) Thickening and tenderness on lateral palpation are signs that a solar keratosis may have developed into invasive squamous cell carcinoma

de Berker et al. [12]	(i) Studies indicate a high spontaneous regression rate in the order of 15–25% for AKs over a 1-year period and a low rate of malignant transformation, less than one in 1,000 per annum. (ii) No therapy or emollient is a reasonable option for mild AKs (iii) Sun block applied twice daily for 7 months may protect against development of AKs (iv) 5-Fluorouracil cream used twice daily for 6 weeks is effective for up to 12 months in clearance of the majority of AKs; due to side-effects of soreness, less aggressive regimens are often used, which may be effective but have not been fully evaluated (v) Cryosurgery is effective for up to 75% of lesions in trials comparing it with photodynamic therapy; it may be particularly superior for thicker lesions but may leave scars

Stockfleth and Kerl (2006) [13]	(i) Cryotherapy is often used and controlled studies are missing. Complete responses differ from 75% to 98%; the recurrence rates of AKs have been estimated from 1.2% to 12% within a 1-year follow-up period (ii) The clinical experience in AK patients receiving MAL-PDT shows complete response rate of 70–78% after a single treatment session and 90% after two treatment sessions one week apart; negative effects of PDT are local pain, risk of photosensitivity (mainly for ALA), and time delay between application of cream and treatment. Photodynamic therapy in comparison to cryotherapy shows significantly better cosmetic results
